# Participation in Breed-Specific Cynological Activities Is Associated with Behavioral Variation in Terrier-Type Dogs: A C-BARQ Study

**DOI:** 10.3390/ani16131976

**Published:** 2026-06-26

**Authors:** Virginia Bellini, Gabriele Stagi, Valentina Gazzano, Rossana Cordon, Angelo Gazzano, Francesca Cecchi, Maria Claudia Curadi, Asahi Ogi

**Affiliations:** 1 Department of Veterinary Sciences, University of Pisa, 56124 Pisa, Italy; virgy.bellini95@gmail.com (V.B.); rossanacvet@gmail.com (R.C.); angelo.gazzano@unipi.it (A.G.); francesca.cecchi@unipi.it (F.C.); maria.claudia.curadi@unipi.it (M.C.C.); 2 Department of Agri-Food Production and Environmental Sciences, University of Firenze, 50144 Firenze, Italy; dr.stagi@gmail.com; 3 Department of Clinical Sciences and Translational Medicine, University of Rome Tor Vergata, 00133 Rome, Italy; asahi.ogi@uniroma2.eu

**Keywords:** C-BARQ, dog behavior, terrier breeds, breed-specific activities, behavioral traits, multivariate analysis

## Abstract

This exploratory study investigated the relationship between demographic, biological, and management-related factors and behavioral traits in 195 terrier-type dogs using the C-BARQ questionnaire. Participation in breed-specific cynological activities (ENCI working trials and aptitude tests) emerged as the most consistent factor: non-participation was associated with higher scores in owner-directed aggression (β = 0.17, *p* = 0.007), non-social fear (β = 0.34, *p* = 0.001), dog-directed fear (β = 0.36, *p* = 0.015), and attachment/attention-seeking (β = 0.32, *p* = 0.026), whereas participation was associated with increased chasing behavior (β = −0.60, *p* < 0.001). Neutered dogs showed higher non-social fear (β = 0.83, *p* < 0.001), touch sensitivity (β = 0.34, *p* = 0.012), and aggression toward conspecifics (β = 0.55, *p* = 0.004) and strangers (β = 0.32, *p* = 0.034). Age primarily influenced energy level (β = −0.09, *p* < 0.001) and dog-directed aggression (β = 0.05, *p* = 0.006) after FDR correction. Sex differences were modest, with males showing higher dog-directed aggression (β = −0.42, *p* = 0.004). Breed contributed to variability in selected domains, with the strongest effects observed for energy level and fear-related traits. Overall, these findings highlight participation in breed-specific activities and neuter status as key factors associated with behavior in terrier-type dogs. These associations are exploratory and require confirmation in future studies.

## Simple Summary

Terrier-type dogs were selectively bred for centuries to hunt small mammals in underground burrows, a functional heritage that shaped their distinctive behavioral profile: high predatory drive, reactivity, tenacity, and a low threshold for frustration. Despite their widespread popularity as companion animals, the behavioral consequences of management practices in this breed group remain poorly understood. This study applied the Canine Behavioral Assessment and Research Questionnaire (C-BARQ) to 195 terrier-type dogs in Italy, examining how demographic and management factors—including age, sex, neuter status, participation in breed-specific cynological activities, and breed—relate to behavioral traits such as aggression, fear, trainability, and attachment. Participation in breed-specific activities emerged as the most consistent factor: dogs not involved in these activities showed higher scores in fear-related traits, and attachment/attention-seeking, while participating dogs displayed stronger chasing behavior, consistent with breed-typical predatory tendencies. Neutered dogs showed elevated fearfulness and aggression toward conspecifics and strangers, raising questions about the association between neuter status and behavior in this group. Breed accounted for variability in energy level and selected fear and social traits, though effects were not uniform across all dimensions. Overall, these findings show that participation in breed-specific activities is associated with behavioral differences in terrier-type dogs. Because the study was cross-sectional, this pattern may reflect both the management context of participating dogs and the tendency of owners to involve dogs already suited to these activities. Even with this caution, the results highlight the importance of considering breed-specific activities, owner selection, and management context when advising terrier owners, breeders, and veterinary practitioners.

## 1. Introduction

The domestication of *Canis lupus familiaris* has produced, through millennia of function-oriented artificial selection, a remarkable diversification of behavioral phenotypes among modern dog breeds [1]. Breed-specific behavioral traits are highly heritable, with over 131 polymorphisms identified in genes expressed in neural tissue [2], and breed-typical predispositions became established before modern breed standards were formalized by the Victorian cynological movement [3]. These innate predispositions persist even in the absence of specific training [4] and define the expected functional profile of each breed group.

However, breed explains only a modest proportion of individual behavioral variance, approximately 9% [3], underscoring the substantial contribution of individual history and environment.

Among breed groups, earth-working Terriers of the Fédération Cynologique Internationale (FCI) Group 3 represent one of the most pronounced cases of functional specialization in domestic canids. Selected for hunting and eliminating small mammals in confined subterranean environments [5], these breeds retain a near-complete predatory motor sequence and morphological characteristics (thoracic circumference below 35 cm, oval rib cage, and flat limb musculature) that directly reflect these functional adaptations [6,7,8]. At the temperamental level, this selective history translates into heightened reactivity, a low predatory threshold, autonomous decision-making, and gameness, encompassing frustration tolerance, high intrinsic motivation, and sustained attentional focus [2,9]. These characteristics may make Terriers vulnerable when they lack appropriate outlets: deprived environments and chronic frustration can promote stereotypies and compulsive disorders [10,11]. Moreover, insufficient physical activity significantly increases the likelihood of repetitive behaviors [12], a concern that extends across domestic dogs broadly, where behavioral problems are reported in 72–85% of individuals [13,14] and aggression accounts for 72.2% of specialist consultations [15].

Terriers are significantly overrepresented among veterinary behavior referrals compared with all other breed groups (*p* < 0.0001; [15]), with elevated aggression prevalence well documented in the literature [16]. These breeds may show compulsive behaviors, such as excessive digging, persistent vocalization, tail chasing, and light or shadow fixation, especially when they lack breed-appropriate outlets [10,11,12].

Beyond these breed-specific challenges, Terriers are not immune to the anxiety-related disorders that affect the broader canine population: 72.5% of dogs display anxiety-related behaviors in at least one context [13], with separation-related disorders affecting 14–20% of individuals [13] and noise phobias being particularly prevalent [17,18]. Indeed, traits such as heightened vigilance and reactivity, which are adaptive in hunting contexts, may become problematic in domestic environments, where they can manifest as hyperreactivity. In addition, early deprivation has been associated with long-term behavioral consequences in earth-working breeds, including defensive aggression, redirected predatory behavior, and handling intolerance [19,20,21,22]. This behavioral risk profile suggests that structured, breed-appropriate outlets may help shape how these dogs behave in domestic settings.

The working trials and aptitude tests for Terriers recognized by the Italian Kennel Club (Ente Nazionale della Cinofilia Italiana, ENCI) provide structured opportunities for these dogs to express behaviors for which they were originally selected. Without appropriate outlets, these same tendencies may manifest as behavior problems [23,24,25]. Participation in breed-specific activities usually reflects a specific management context. Dogs are regularly exposed to breed-relevant stimuli and engage in structured activities with their handlers. This repeated cooperation may strengthen the dog–owner bond and support cooperation and emotional stability [26,27]. Dogs from working lines may also display behavioral profiles that differ systematically from those of conspecifics from show or companion lines [16,28].

Empirical evidence suggests that active management and regular engagement in activities may have a protective association with anxiety-related behaviors in dogs. Large-scale surveys have shown that infrequent participation in activities or training is significantly associated with elevated non-social fearfulness and fear of strangers and conspecifics [13,29,30], and sporting activity has been reported as a factor associated with improvement in generalized anxiety [31]. If a lack of breed-appropriate outlets increases the risk of behavior problems [25,32,33], it follows that dogs engaged in breed-specific morpho-functional assessments may display more favorable behavioral profiles compared to conspecifics maintained exclusively as companions, a hypothesis that has not, however, been examined systematically in terrier-type dogs using standardized psychometric instruments.

To address this gap, the present study applied the Canine Behavioral Assessment and Research Questionnaire (C-BARQ; [34]), in the full 100-item Italian C-BARQ translation validated by Broseghini and colleagues [35], to a sample of terrier-type dogs varying in their level of involvement in breed-specific cynological activities. The C-BARQ quantifies behavioral responses in everyday situations via standardized owner reports, has been shown to be less susceptible to breed stereotyping than expert-based assessments [36], and has already been employed in the Italian cynological context to characterize breed-specific behavioral profiles [37,38,39,40].

The behavioral dimensions captured by the instrument, aggression, fear and anxiety, compulsive behaviors, excitability, and trainability, correspond directly to the areas of greatest clinical relevance for this breed group. We hypothesized that dogs with higher levels of involvement in breed-specific cynological activities would display lower scores in fear- and anxiety-related dimensions and in owner-directed aggression, and higher scores in trainability, compared to conspecifics maintained exclusively as companions. Breed, age, sex, and neuter status were included as covariates, with effects expected to be consistent with those reported in the broader canine behavioral literature.

The aim of the present study was therefore to evaluate whether participation in breed-specific morpho-functional assessments is associated, in terrier-type dogs, with more favorable behavioral profiles across the dimensions captured by the C-BARQ.

## 2. Materials and Methods

This cross-sectional study employed a voluntary and anonymous sampling strategy. The questionnaire was administered online through social media platforms and breed club pages and was further disseminated among breeders and owners by word of mouth and through direct collaboration with the Italian Terrier Society (SIT), which actively supported distribution of the survey among its members and their wider network of owners and breeders. All participants were informed about the general aims of the study and their right to anonymity. The questionnaire was administered anonymously via an online platform; no personal identifiable information was collected. Prior to participation, respondents were informed of the general aims of the study and of the anonymous nature of data collection. Digital informed consent was obtained through an explicit agreement checkbox displayed at the beginning of the questionnaire, which participants were required to confirm before proceeding. Because participation was voluntary and fully anonymous, and no sensitive personal data were collected, formal written informed consent was not required beyond this digital confirmation. Completion of the survey required approximately 10–15 min. All questionnaire items required a mandatory response before submission. Eligibility criteria included dogs aged at least one year and a minimum cohabitation period of six months with the owner, in order to ensure that respondents had sufficient observational experience to provide a reliable assessment of the dog’s typical behavioral profile [34]. Questionnaires were excluded from the analysis if they were incomplete, displayed inconsistent or evidently random response patterns, referred to dogs younger than one year of age at the time of completion, or involved dogs that had lived with the owner for less than six months. In addition, dogs presenting with ongoing medical conditions that could potentially influence behavioral expression, such as neurological, endocrine, or severe orthopedic disorders, or other documented conditions associated with direct or indirect behavioral alterations, were excluded. Exclusion on medical grounds was based on responses to the relevant item in the demographic section, in order to minimize potential confounding effects arising from behavior changes secondary to underlying medical conditions. Owners who owned more than one eligible dog were asked to complete the questionnaire for only one animal, to avoid potential clustering effects.

Behavioral assessment was performed using the C-BARQ [34], a standardized owner-report instrument comprising 100 items organized into seven broad sections: Trainability, Aggression, Fear, Separation-Related Behaviors, Excitability, Attachment/Attention-Seeking, and Miscellaneous, which yield 15 behavioral subscales, following the scoring procedure described by Serpell and Duffy. The Aggression section is divided into four subscales: Stranger-Directed Aggression, Owner-Directed Aggression, Dog Rivalry, and Dog-Directed Aggression. The Fear section is divided into four subscales: Stranger-Directed Fear, Non-Social Fear, Dog-Directed Fear, and Touch Sensitivity. The remaining five sections, Trainability, Separation-Related Behaviors, Excitability, Attachment/Attention-Seeking, and Miscellaneous, each correspond to a single sub-scale, with the Miscellaneous section further comprising three additional subscales: Chasing, Energy Level, and Miscellaneous Behaviors. Depending on the subscale, items are scored either on a six-point frequency scale (0 = not applicable, 1 = never, 5 = always) or on a five-point intensity scale (0 = none, 4 = severe), with higher scores indicating more problematic behaviors, except for the Trainability subscale where higher scores reflect more desirable traits.

The Italian translation validated by Broseghini and colleagues [35] was employed; this version showed acceptable to good internal reliability across most subscales (Cronbach’s α ranging from 0.60 to 0.92) and a factor structure broadly consistent with the original instrument.

Behavioral dimension scores were computed as the mean of the corresponding subscale items and treated as continuous variables for statistical analysis, in line with established practice in C-BARQ-based research [16,34,41,42]. Internal consistency of each subscale was additionally assessed in the present sample using Cronbach’s alpha coefficient.

Statistical analyses were conducted using Jamovi (version 2.6; The Jamovi project, 2024).

Prior to computing subscale means, zero responses were handled differently depending on their meaning within each behavioral dimension. For dimensions in which a response of zero indicated that the behavior was not applicable to the dog (trainability, chasing, separation-related behaviors, excitability, attachment/attention-seeking, energy level, and miscellaneous behaviors), zero responses were treated as missing values and excluded from mean computation. For dimensions in which zero indicated the absence or minimum intensity of the behavior (aggression- and fear-related dimensions, and touch sensitivity), zero responses were retained in the analysis.

Associations between demographic and management variables and C-BARQ behavioral scores were assessed using multiple linear regression models. Each C-BARQ scale was analyzed separately as a dependent variable.

The following predictors were included in all models: age (continuous), sex, neuter status, participation status, and breed (categorical variables). Jack Russell Terrier was used as the reference category for breed. Breeds represented by smaller numbers were grouped into the “other terrier breeds” category to retain these dogs in the analysis while avoiding sparse breed-specific contrasts. No formal minimum sample-size threshold was applied.

For the purposes of the present study, dogs were classified as participating or non-participating based on owner-reported involvement in ENCI-recognized breed-specific cynological activities. Dogs were classified as participating if owners reported regular, ongoing involvement in ENCI-recognized working trials or aptitude tests at the time of data collection (mean = 1.97 sessions/month, SD = 1.81). Dogs were classified as non-participating if no such involvement was reported.

This variable therefore refers to structured participation in breed-typical work and to the management context that usually accompanies it, including regular exposure to breed-relevant stimuli and cooperative dog–handler activity. It does not measure physical exertion, exercise volume, daily movement, or environmental enrichment. Accordingly, dogs engaged only in non-breed-specific disciplines, such as agility or obedience, were classified as non-participating. Classification was based on regular ongoing participation, not on a frequency threshold; the reported mean describes participation intensity in the participating group and was not used as a cut-off.

Model assumptions were evaluated by inspection of residuals. Model fit was assessed using R^2^ and adjusted R^2^ values, and overall model significance was evaluated using F-tests. Regression coefficients (β), representing the expected change in the behavioral score associated with each predictor, along with corresponding standard errors and *p*-values, are reported. For participation status, positive β values indicate higher scores in non-participating dogs, and negative β values indicate higher scores in participating dogs. Standardized regression coefficients (β-std) were also reported to facilitate comparison of effect sizes across predictors.

**Figure 1:**
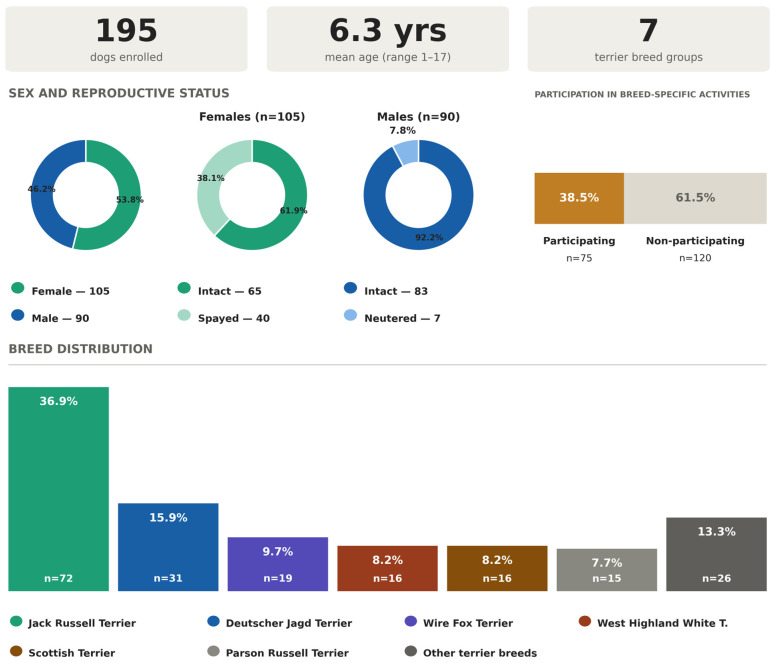
Demographic characteristics of the study population.

Statistical significance was set at *p* < 0.05. To account for multiple comparisons, two complementary corrections were applied according to the nature of the analyses. For the regression coefficients, which tested a large number of associations across behavioral dimensions, the Benjamini–Hochberg false discovery rate (FDR) procedure was used, as it controls the expected proportion of false positives and is appropriate for analyses of an exploratory nature; FDR-adjusted *p*-values (q-values) are reported alongside the uncorrected values, and associations with q < 0.05 were considered robust. For the post hoc breed pairwise comparisons, a Bonferroni correction was applied, this being the conventional family-wise approach for a restricted set of planned post hoc contrasts. Figure 1 was created with the assistance of Claude (Sonnet 4.5, Anthropic, San Francisco, CA, USA), a large language model-based artificial intelligence tool.

## 3. Results

A total of 219 questionnaires were initially collected. Of these, 24 were excluded due to the presence of active diseases at the time of assessment. A total of 195 questionnaires were included in the final analysis.

The demographic characteristics of the study population are summarized in Figure 1, including the distribution of dogs by sex, neuter status, participation status, and breed group. Briefly, the sample included 105 females and 90 males; 75 dogs were classified as participating in ENCI-recognized breed-specific activities and 120 as non-participating. Breed groups were unevenly represented, with Jack Russell Terrier forming the largest group (n = 72) and the remaining groups ranging from 15 to 31 dogs, plus a heterogeneous “other terrier breeds” category (n = 26).

Descriptive statistics for all behavioral dimensions are reported in Table 1. The highest mean scores were observed for “Chasing behavior”, “Trainability”, “Attachment/attention-seeking”, and “Energy level”. At the lower end of the distribution, “Aggression toward owner” and “Fear of strangers” recorded the lowest mean values. Internal consistency was acceptable to good across most behavioral dimensions (Cronbach’s α ranging from 0.573 to 0.989), except for “Trainability”, which showed lower reliability (α = 0.573), likely reflecting the heterogeneous and context-specific nature of the items.

Multiple linear regression models were used to explore associations between demographic and management factors and C-BARQ behavioral scores. Several behavioral domains were significantly associated with the predictors considered, although the strength and consistency of these associations varied across scales.

Among the predictors, participation status and neuter status emerged as the most consistent factors associated with behavioral variation. Non-participation in breed-specific activities was associated with higher scores in several behavioral domains, particularly owner-directed aggression, fear-related traits, and attention-seeking. In contrast, participation in breed-specific activities was associated with increased chasing behavior.

Neuter status was also associated with multiple behavioral traits, particularly those related to fear and aggression, with neutered dogs generally showing higher scores across these domains.

Age showed more limited and domain-specific effects, primarily influencing energy levels and, to a lesser extent, dog-directed aggression.

Sex differences were relatively modest and mainly observed in behaviors directed toward other dogs.

Detailed results of the regression analyses are presented in Table 2 and Table 3. Non-significant predictors for all behavioral dimensions are reported in Supplementary Table S1.

Standardized coefficients showed that neuter status and participation status exerted the largest effects across behavioral domains, particularly for non-social fear (β-std = 1.14 and β-std = 0.47, respectively) and chasing behavior (β-std = −0.57).

The sample included Jack Russell Terrier (n = 72), which represented the largest breed group and was therefore used as the reference category, Deutscher Jagdterrier (n = 31), Fox Terrier (n = 19), West Highland White Terrier (16), Scottish Terrier (16), Parson Russell Terrier (n = 15), and other terrier breeds (n = 26). The “other terrier breeds” category comprised Cairn Terrier (n = 8), Irish Terrier (n = 6), Border Terrier (n = 3), English Toy Terrier (n = 2), Smooth Fox Terrier (n = 1), Norfolk Terrier (n = 1), Brazilian Terrier (n = 1), Bedlington Terrier (n = 1), Boston Terrier (n = 1), Irish Soft Coated Wheaten Terrier (n = 1), and Welsh Terrier (n = 1). Because several breed groups were small and unevenly represented, and because the “other terrier breeds” category was heterogeneous, breed contrasts and pairwise comparisons should be considered exploratory and unevenly powered.

Breed contributed to variability in several models, with significant main effects observed in energy level (F = 3.57, *p* = 0.001), trainability (F = 2.34, *p* = 0.033), fear of strangers (F = 2.52, *p* = 0.023), attachment/attention-seeking (F = 2.59, *p* = 0.014), aggression toward strangers (F = 2.16, *p* = 0.049), and miscellaneous behaviors (F = 2.04, *p* = 0.012), as summarized in Table 4; the complete breed effect across all models, including non-significant scales, is provided in Supplementary Table S2. Post hoc comparisons revealed specific pairwise differences between breeds in selected domains (Table 5), particularly for energy level and trainability; non-significant breed comparisons are reported in Supplementary Table S3. However, significant pairwise differences were not observed across all behavioral dimensions where breed was significant, suggesting that the overall breed effect reflects a distributed pattern of variation rather than strong contrasts between specific pairs.

Several behavioral traits, including excitability, separation-related behaviors, trainability, and dog rivalry, were not significantly associated with any of the predictors included in the models.

## 4. Discussion

The present study examined behavioral traits in terrier-type dogs in relation to demographic, biological, and management-related factors using the C-BARQ questionnaire. Participation in breed-specific cynological activities and neuter status emerged as the most consistent factors associated with behavior across multiple C-BARQ dimensions.

Given the cross-sectional design and the number of dimensions examined, these analyses are exploratory and hypothesis-generating. The models explained a moderate proportion of behavioral variation, with R^2^ values mostly ranging from approximately 0.10 to 0.20 and reaching the highest value for non-social fear (R^2^ = 0.282). This indicates that the predictors included in the models captured only part of the behavioral variation observed in the sample. Behavioral traits are likely influenced by additional factors not measured here, including training history, owner management style, early experience, living environment, and genetic relatedness. The results should therefore be interpreted as partial associations rather than as comprehensive explanatory models of terrier behavior.

The descriptive data showed a behavioral profile consistent with the selective history of earth-working terriers. The highest mean scores were recorded for chasing behavior, trainability, attachment/attention-seeking, and energy level, reflecting the predatory drive, cooperative demands, and owner-oriented behavioral tendencies characteristic of FCI Group 3 breeds [5,16], while the elevated trainability scores may additionally reflect the active management context of a sample largely composed of owners engaged with breed clubs and cynological associations [43].

A key finding of this study is that participation in breed-specific activities was associated with several behavioral domains, particularly fear-related traits and attachment/attention-seeking. Although owner-directed aggression was associated with participation status in the unadjusted analysis, this association did not remain significant after correction for multiple comparisons.

The largest activity-related effect was observed for chasing behavior (β-std = −0.57), followed by non-social fear (β-std = 0.47) and attachment/attention-seeking behavior (β-std = 0.42). Two non-exclusive explanations may account for this pattern. First, participation in breed-specific cynological activities may reflect a management context that includes structured work, repeated dog–handler cooperation, and exposure to varied environments. With respect to fear-related traits, this interpretation is broadly consistent with large-scale studies reporting associations between lower activity or reduced training and higher fearfulness in dogs [29,30]. Second, and equally plausibly, owners may be more likely to enter dogs with lower fearfulness and lower attention-seeking into working trials or aptitude tests. The cross-sectional design of the present study cannot distinguish between these explanations. Therefore, the welfare implications of participation should be interpreted as hypotheses to be tested, rather than as evidence of a protective effect.

The association between participation and increased chasing behavior likely reflects the expression of breed-typical predatory tendencies, which breed-specific trials actively assess and reward, rather than a maladaptive outcome. Together, these findings suggest that structured participation in breed-specific activities may be relevant to the management and welfare of terrier-type dogs, particularly when interpreting behavioral traits linked to fear, attachment, and predatory motivation.

The lower attachment/attention-seeking scores observed in participating dogs may reflect the management context associated with breed-specific activities. Dogs involved in these activities are often exposed to varied environments and repeated dog–handler interactions, which may support greater autonomy and reduce attention-seeking behavior. However, the present study did not directly measure independence, owner management style, exercise volume, or physical and mental stimulation; therefore, this interpretation remains tentative. This cautious interpretation is consistent with previous evidence of linking activity, environmental conditions, and management-related factors with behavioral outcomes in dogs [12,25,44].

The association between participation and higher chasing scores, far from representing a paradox, reflects the fact that working trials specifically assess and reward drive, voice, and perseverance in earth work [23,24], creating a management context that selectively reinforces the expression of predatory behavioral components, precisely those most heritable and selection-responsive in dogs [45].

Sex differences were relatively limited, suggesting that sex may play a less prominent role compared to other variables such as participation status or neuter status. The sex effect on aggression toward dogs was driven by higher scores in males, concordant with previous reports of elevated inter-dog aggression in intact male dogs, though the direction of this effect can vary across samples depending on neuter status distribution [1]. The overall limited contribution of sex, with no significant effects on aggression toward strangers or owner, chasing, or trainability, is consistent with large-scale evidence indicating that sex accounts for a smaller proportion of behavioral variance than breed membership or management context [3]. As neuter status was not evenly distributed between sexes (with intact dogs more common among males and neutered among females) residual confounding cannot be entirely excluded despite simultaneous inclusion of both variables in the models, and sex-related findings should be interpreted with appropriate caution.

Neuter status was associated with several fear- and aggression-related traits. Neutered dogs showed higher scores for touch sensitivity, non-social fear, aggression toward conspecifics, and aggression toward strangers. The standardized coefficients further suggest that the association between neuter status and non-social fear was among the strongest effects detected in the study (β-std = 1.13). These results should be interpreted as associations with neuter status, not as evidence that neutering caused increased fearfulness or aggression. These findings are consistent with previous studies reporting associations between neuter status, fearfulness, and reactivity in some dogs [46,47,48,49]. Large C-BARQ datasets have also linked reduced lifetime exposure to gonadal hormones with higher fearfulness and, in some populations, higher aggression [50]. Similar breed-related patterns have been reported for boldness across multiple breed groups [51], and, more recently, in Australian Shepherds [40].

These findings should be interpreted strictly as associations with neuter status, not as evidence that neutering caused higher fearfulness or aggression. Their interpretation is further complicated by the distribution of neuter status in the present sample: neutered dogs were predominantly female (40 spayed females versus 7 neutered males), making neuter status and female sex nearly collinear. Although both variables were included in the models, the elevated fear- and aggression-related scores associated with neuter status cannot be cleanly separated from a possible sex effect and should therefore be interpreted with caution.

Reverse causality is also possible. Dogs with pre-existing fearfulness, reactivity, or aggression may have been more likely to be neutered by their owners. This is particularly relevant because neutering age and behavioral history before neutering were not recorded.

Age-related effects were generally consistent with expectations and previous studies [52], with older dogs showing lower energy levels but an increased level of conspecific aggression.

Breed was included as a control variable and was associated with several behavioral traits. However, these effects should be interpreted cautiously. Jack Russell Terriers formed the largest group, whereas several other breeds were represented by smaller subsamples or grouped within the heterogeneous “other terrier breeds” category. In addition, unmeasured relatedness among dogs may have contributed to similarities within breed groups. Therefore, the observed breed effects should be viewed as exploratory patterns rather than definitive breed-level differences. The lower trainability scores observed in Deutscher Jagd Terriers compared with Jack Russell Terriers may reflect differences in selection history and working style among terrier subtypes. However, given the small and uneven breed sample sizes, this interpretation should be considered exploratory. This interpretation is consistent with evidence that breed explains only a modest proportion of individual behavioral variance, and that environmental context and individual history also play important roles [53]. The discrepancy between the significant main effects of breed and the more limited number of significant pairwise comparisons likely reflect distributed variability across multiple breeds rather than strong differences between specific pairs, highlighting the complexity of breed-related behavioral variation.

Some behavioral traits, such as excitability and separation-related behaviors, were not significantly associated with the variables included in the models. This suggests that these dimensions may be influenced by other factors not captured in the present study, such as training history, owner behavior, or individual temperament.

Taken together, the results highlight the importance of considering both environmental and biological factors when evaluating canine behavior. In particular, participation in breed-specific cynological activities emerges as a key management-related factor associated with multiple behavioral outcomes, suggesting potential implications for management and welfare in terrier-type dogs.

This study has several limitations that should be considered when interpreting the results.

First, the cross-sectional design precludes causal inference regarding the relationship between participation in breed-specific cynological activities and behavioral outcomes. The observed associations are consistent with a possible protective association of participation but cannot exclude the alternative interpretation that dogs with more favorable behavioral profiles are more likely to be enrolled in working trials and aptitude tests by their owners, a selection effect that would produce the same pattern of results without any causal influence of participation on behavior. Longitudinal designs tracking behavioral trajectories before and after engagement with cynological activities would be required to address this question.

Second, reliance on owner-reported questionnaire data introduces the possibility of response bias. Owners engaged in breed clubs and working activities may be systematically more attentive to their dogs’ behavior, potentially producing more accurate assessments; alternatively, they may be motivated to report more favorable profiles for their animals, introducing upward bias on dimensions such as trainability and downward bias on fear and aggression. Recruitment through social media platforms, breed club networks, and the SIT likely attracted a self-selected population of highly engaged and motivated owners, which may have contributed to the generally low fear and aggression scores observed and limits the generalizability of the findings to the broader terrier-owning population. Although the C-BARQ has demonstrated satisfactory psychometric properties and reduced susceptibility to breed stereotyping compared with expert-based assessments [35,36], owner report bias remains an inherent limitation of this methodology.

Third, while the models included key demographic and management variables, other potentially relevant factors were not assessed and may have influenced the observed behaviors. In particular, classification as participating is likely correlated with a broader set of owner- and management-related characteristics—including owner engagement, training experience, breeder involvement, management quality, and possibly socioeconomic factors—none of which were measured here. The associations attributed to participation may therefore partly reflect this wider owner-related context rather than participation in breed-specific activities per se. Such unmeasured factors include training methods and intensity, for which owner and handler experience is particularly relevant in a sample enriched for cynological engagement; living environment and access to outdoor space, which may partially confound the association between participation and behavior given the likely correlation between working trial participation and housing conditions; and daily routines. Moreover, the sample size, while sufficient for the analyses conducted, limits the power to detect small effects and may not adequately represent the full range of behavioral variation within each terrier breed group. The relatively small within-breed samples for less common breeds, grouped under the ‘other terrier breeds’ category, preclude breed-level analyses that would be required to identify the specific within-group contrasts driving the observed breed group effects. Larger, breed-specific studies would be required to decompose these effects and to examine the moderating role of specific breed characteristics, such as degree of functional specialization or lineage (working versus show line).

Fourth, the kinship among subjects was not formally assessed. This concern is particularly salient in the present sample, in which the Jack Russell Terrier, a numerically widespread breed at the national level, accounts for the majority of subjects (n = 72), while all remaining breeds are represented by substantially smaller subsamples reflecting their more restricted breeding populations in Italy. In purebred dogs generally, small census sizes are associated with reduced effective population sizes and elevated relatedness among registered individuals, with most breeds exhibiting inbreeding effective population sizes well below 100 regardless of census size [54]. Recruitment through breed-specific networks and the SIT may therefore have resulted in an overrepresentation of related individuals, such that behavioral similarities within breed groups could partly reflect shared breeding lines rather than the effects of the variables examined. Future studies should account for pedigree structure through mixed models incorporating a genetic relatedness matrix.

Fifth, age at neutering was not recorded, representing a potential source of residual confounding, as associations between neuter status and behavior may vary depending on the age at which neutering is performed [55]. Future research should systematically record this information to allow a more refined assessment of neuter-related behavioral effects.

Finally, although FDR and Bonferroni corrections were applied, the possibility of residual false-positive findings cannot be completely excluded given the exploratory nature of the study.

Future research should address these limitations through longitudinal designs and objective behavioral assessments complementing owner-report instruments, alongside the incorporation of genetic markers and pedigree data to allow direct estimation of heritability and relatedness effects.

## 5. Conclusions

The present study provides evidence that behavioral variation in terrier-type dogs is associated with biological factors and participation in breed-specific cynological activities. Participation status was associated with several C-BARQ dimensions, whereas neuter status was associated mainly with fear- and aggression-related traits. These findings support the importance of considering both biological factors and management context when evaluating behavior in terrier-type dogs. The domain-specific effects of neuter status further underscore the importance of individualized assessment in clinical practice. Future research using longitudinal designs, objective behavioral assessments, and genetic or pedigree data would help clarify whether these associations reflect causal effects, selection effects, or shared genetic background.

## Figures and Tables

**Table 1:** Descriptive statistics for behavioral dimensions in terrier-type dogs (M = mean, SD = standard deviation).

Behavioral Dimension	Mean	SD	Cronbach’s α
Trainability	3.53	0.48	0.573
Aggression toward strangers	0.71	0.75	0.895
Aggression toward owner	0.15	0.40	0.836
Aggression toward dogs	1.58	1.00	0.797
Dog rivalry	0.61	0.92	0.893
Fear of strangers	0.42	0.79	0.909
Non-social fear	0.77	0.73	0.676
Dog directed fear	0.75	0.92	0.823
Separation-related behaviors	1.48	0.52	0.974
Excitability	2.34	0.84	0.981
Attachment/attention-seeking	3.25	0.90	0.976
Touch sensitivity	0.64	0.67	0.687
Energy level	3.20	1.16	0.984
Chasing behavior	3.79	1.02	0.962
Miscellaneous behaviors	1.65	0.36	0.989

**Table 2:** Model summary of linear regression analyses.

C-BARQ Scale	R^2^	Adj. R^2^	F	*p*-Value
Aggression toward strangers	0.099	0.073	2.45	0.032
Aggression toward owner	0.095	0.068	2.31	0.045
Aggression toward dogs	0.191	0.166	7.62	<0.001
Chasing behaviors	0.164	0.139	6.45	<0.001
Energy level	0.225	0.202	9.75	<0.001
Non-social fear	0.282	0.261	13.54	<0.001
Dog-directed fear	0.106	0.080	2.67	0.032
Attachment /attention-seeking	0.133	0.107	3.92	0.005
Touch sensitivity	0.121	0.095	3.11	0.012

Only models reaching statistical significance (*p* < 0.05) are shown.

**Table 3:** Significant predictors from linear regression models.

Scale	Predictor	β	β-std	SE	95% CI	*p*-Value	FDR-BH *p*-Value	Higher in
Aggression toward strangers	Neuter	0.32	0.467	0.15	0.02–0.62	0.034	0.034	Neutered dogs
Aggression toward owner	Participation status	0.17	0.34	0.06	0.05–0.29	0.007	0.0105	Non-participating dogs
Aggression toward dogs	Age	0.05	0.22	0.02	0.01–0.09	0.006	0.0103	Older dogs
Aggression toward dogs	Sex	−0.42	0.42	0.15	−0.71–−0.14	0.004	0.008	Males
Aggression toward dogs	Neuter	0.55	0.52	0.19	0.18–0.92	0.004	0.008	Neutered dogs
Chasing behaviors	Participation status	−0.60	−0.57	0.14	−0.88–−0.32	<0.001	0.003	Participating dogs
Energy level	Age	−0.09	−0.29	0.01	−0.11–−0.06	<0.001	0.003	Younger dogs
Non-social fear	Neuter	0.83	1.13	0.17	0.49–1.17	<0.001	0.003	Neutered dogs
Non-social fear	Participation status	0.34	0.47	0.10	0.14–0.54	0.001	0.003	Non-participating dogs
Dog directed fear	Participation status	0.30	0.32	0.15	0.07–0.65	0.015	0.018	Non-participating dogs
Attachment /attention-seeking	Participation status	0.32	0.42	0.14	0.04–0.59	0.026	0.0284	Non-participating dogs
Touch sensitivity	Neuter	0.34	0.42	0.13	0.08–0.61	0.012	0.016	Neutered dogs

Note: For participation status, positive β values indicate higher scores in non-participating dogs, and negative β values indicate higher scores in participating dogs.

**Table 4:** Main effect of breed across models.

C-BARQ Scale	F (Breed)	*p*-Value
Trainability	2.34	0.033
Aggression toward strangers	2.16	0.049
Fear of strangers	2.52	0.023
Attachment/attention-seeking	2.59	0.014
Miscellaneous behaviors	2.04	0.012
Energy level	3.57	0.001

**Table 5:** Pairwise breed comparisons reported in post hoc analyses.

Comparison	Scale	β	*p*-Value	Bonferroni-Adjusted *p*-Value	Higher in
Deutscher Jagd Terrier vs. Jack Russell	Trainability	−0.34	0.002	0.042	Jack Russell
Deutscher Jagd Terrier vs. Jack Russell	Aggression toward owner	0.18	0.044	0.924	Deutscher Jagd Terrier
Parson Russell vs. Jack Russell	Fear of strangers	0.58	0.001	0.021	Parson Russell
Deutscher Jagd Terrier vs. Jack Russell	Energy level	3.88	<0.001	0.021	Deutscher Jagd Terrier
Parson Russell vs. Jack Russell	Energy level	3.58	0.002	0.042	Parson Russell
Fox Terrier vs. Jack Russell	Energy level	3.68	<0.001	0.021	Fox Terrier

## Data Availability

The de-identified dataset supporting the findings of this study is available as Supplementary Material accompanying this article.

## Supplementary Materials

The following supporting information can be downloaded at: https://www.mdpi.com/article/10.3390/ani16131976/s1, Table S1. Non-significant predictors from multiple linear regression models. Table S2. Main effect of breed across models. Table S3. Non-significant breed comparisons. Table S4: De-identified dataset.
