# Supplementary material for: Participation in Breed-Specific Cynological Activities Is Associated with Behavioral Variation in Terrier-Type Dogs: A C-BARQ Study
Source: Animals (Basel). 2026 Jun 26;16(13):1976. doi: 10.3390/ani16131976 (PMC13359903; doi:10.3390/ani16131976)
Supplement: Supplementary file 1 [file animals-16-01976-s001.zip › Table S1_CBARQ_supplementary_non_significant_full.pdf]

Supplementary Table S1. Non-significant predictors from multiple linear regression models

Scale	Predictor	Beta	SE	95% CI	p-value
Energy level	Sex	-0.284	0.168	-0.616 to 0.047	0.092
Energy level	Neuter status	-0.224	0.224	-0.665 to 0.218	0.318
Energy level	Activity level	0.321	0.176	-0.026 to 0.668	0.069
Chasing	Age	-0.031	0.020	-0.070 to 0.008	0.118
Chasing	Sex	0.162	0.150	-0.135 to 0.459	0.283
Chasing	Neuter status	-0.070	0.200	-0.465 to 0.326	0.729
Miscellaneous	Age	-0.010	0.007	-0.024 to 0.004	0.154
Miscellaneous	Sex	-0.033	0.053	-0.138 to 0.072	0.538
Miscellaneous	Activity level	0.059	0.056	-0.052 to 0.169	0.295
Trainability	Age	-0.005	0.010	-0.024 to 0.014	0.604
Trainability	Sex	-0.038	0.074	-0.183 to 0.107	0.607
Trainability	Neuter status	-0.060	0.098	-0.254 to 0.134	0.540
Trainability	Activity level	-0.107	0.077	-0.258 to 0.045	0.166
Stranger-directed aggression	Age	0.002	0.015	-0.027 to 0.031	0.895
Stranger-directed aggression	Sex	-0.144	0.114	-0.368 to 0.080	0.207
Stranger-directed aggression	Activity level	-0.133	0.118	-0.367 to 0.101	0.263
Owner-directed aggression	Age	-0.005	0.008	-0.021 to 0.011	0.535
Owner-directed aggression	Sex	0.023	0.060	-0.097 to 0.142	0.709
Owner-directed aggression	Neuter status	0.159	0.081	-0.000 to 0.318	0.050
Dog-directed aggression	Activity level	-0.292	0.150	-0.588 to 0.005	0.054
Dog rivalry	Age	0.009	0.018	-0.028 to 0.045	0.643
Dog rivalry	Sex	0.272	0.140	-0.004 to 0.548	0.054
Dog rivalry	Neuter status	0.283	0.187	-0.086 to 0.651	0.132

Dog rivalry	Activity level	-0.242	0.146	-0.530 to 0.046	0.099
Fear of strangers	Sex	-0.186	0.116	-0.415 to 0.043	0.111
Fear of strangers	Activity level	0.204	0.121	-0.035 to 0.442	0.094
Dog-directed fear	Age	-0.008	0.018	-0.045 to 0.028	0.654
Dog-directed fear	Neuter status	0.198	0.187	-0.170 to 0.566	0.290
Separation-related behavior	Age	-0.007	0.011	-0.028 to 0.014	0.503
Separation-related behavior	Sex	0.124	0.080	-0.033 to 0.282	0.122
Separation-related behavior	Neuter status	-0.027	0.106	-0.237 to 0.183	0.799
Excitability	Age	-0.030	0.017	-0.064 to 0.004	0.079
Excitability	Sex	-0.001	0.130	-0.257 to 0.255	0.993
Excitability	Neuter status	0.035	0.173	-0.306 to 0.376	0.840
Excitability	Activity level	0.267	0.136	-0.001 to 0.535	0.051
Attachment/attention-seeking	Age	-0.008	0.018	-0.043 to 0.027	0.655
Attachment/attention-seeking	Sex	-0.168	0.134	-0.433 to 0.096	0.211
Attachment/attention-seeking	Neuter status	-0.054	0.178	-0.406 to 0.298	0.763
Touch sensitivity	Age	0.001	0.013	-0.026 to 0.027	0.947
Touch sensitivity	Sex	0.091	0.101	-0.109 to 0.291	0.372
Touch sensitivity	Activity level	0.131	0.106	-0.078 to 0.341	0.217

Note. Only non-significant demographic and management predictors are shown ($p >= 0.05$). Non-significant breed contrasts are not displayed to reduce table length. Beta = unstandardized regression coefficient; SE = standard error; CI = confidence interval. Direction is descriptive only and should not be interpreted as statistically supported. For categorical predictors, direction reflects the coding used in the models; for activity level, positive coefficients indicate higher scores in dogs with lower activity, whereas negative coefficients indicate higher scores in active dogs.
