# Supplementary material for: Participation in Breed-Specific Cynological Activities Is Associated with Behavioral Variation in Terrier-Type Dogs: A C-BARQ Study
Source: Animals (Basel). 2026 Jun 26;16(13):1976. doi: 10.3390/ani16131976 (PMC13359903; doi:10.3390/ani16131976)
Supplement: Supplementary file 1 [file animals-16-01976-s001.zip › Table S2_ breed effect all models.pdf]

Supplementary Table S2. Main effect of breed across models

C-BARQ scale	F (breed)	p-value
Trainability	2.34	0.033
Stranger-directed aggression	2.16	0.049
Owner-directed aggression	1.06	0.390
Dog-directed aggression	1.84	0.093
Dog rivalry	1.22	0.299
Fear of strangers	2.52	0.023
Non-social fear	0.68	0.667
Dog-directed fear	1.32	0.244
Separation-related behaviors	1.26	0.270
Excitability	0.87	0.530
Attachment/attention-seeking	2.59	0.014
Touch sensitivity	1.69	0.110
Miscellaneous behaviors	2.04	0.012
Energy level	3.57	0.001

Note: F and p-values refer to the main effect of breed (Type III sums of squares). Trends are defined as $0.05 \le p < 0.10$.
