# Supplementary material for: Participation in Breed-Specific Cynological Activities Is Associated with Behavioral Variation in Terrier-Type Dogs: A C-BARQ Study
Source: Animals (Basel). 2026 Jun 26;16(13):1976. doi: 10.3390/ani16131976 (PMC13359903; doi:10.3390/ani16131976)
Supplement: Supplementary file 1 [file animals-16-01976-s001.zip › Table S3_nonsignificant breed comparison.pdf]

Supplementary Table S3. Non-significant breed comparisons

Comparison	Scale	p-value
Most breed contrasts	Multiple scales	>0.05
Dog rivalry breed contrasts	Dog rivalry	>0.05
Excitability breed contrasts	Excitability	>0.05
