# Supplementary material for: Participation in Breed-Specific Cynological Activities Is Associated with Behavioral Variation in Terrier-Type Dogs: A C-BARQ Study
Source: Animals (Basel). 2026 Jun 26;16(13):1976. doi: 10.3390/ani16131976 (PMC13359903; doi:10.3390/ani16131976)
Supplement: Supplementary file 1 [file animals-16-01976-s001.zip › Table S4_DATA_SET.pdf]

BREED	AGE	SEX	CASTRATION
Jack Russell Terrier	2	M	Intact
Deutscher Jagd Terrier	17	F	Neutered
Parson Russell Terrier	4	M	Intact
Parson Russell Terrier	11	F	Neutered
Parson Russell Terrier	5	F	Intact
Deutscher Jagd Terrier	10	F	Neutered
Jack Russell Terrier	3	M	Intact
Jack Russell Terrier	12	F	Intact
Jack Russell Terrier	4	M	Intact
Deutscher Jagd Terrier	8	M	Intact
Deutscher Jagd Terrier	4	M	Intact
Jack Russell Terrier	6	M	Intact
Fox Terrier a pelo duro	10	F	Intact
Deutscher Jagd Terrier	2	M	Intact
Jack Russell Terrier	3	M	Intact
Jack Russell Terrier	2	M	Intact
Fox Terrier a pelo duro	7	F	Intact
Jack Russell Terrier	6	F	Neutered
Jack Russell Terrier	4	M	Intact
Jack Russell Terrier	5	M	Intact
West Highland White Terrier	5	F	Intact
Fox Terrier a pelo duro	4	M	Intact
Deutscher Jagd Terrier	4	M	Intact
Fox Terrier a pelo duro	4	F	Intact
Fox Terrier a pelo duro	4	F	Intact
Jack Russell Terrier	6	M	Intact
Jack Russell Terrier	12	M	Intact
Fox Terrier a pelo duro	2	F	Intact
Fox Terrier a pelo duro	5	M	Intact
Scottish Terrier	7	M	Intact
Border Terrier	3	F	Intact
West Highland White Terrier	7	F	Intact
Norfolk terrier	2	F	Intact
Cairn Terrier	17	F	Intact
Fox Terrier a pelo duro	8	M	Intact
Jack Russell Terrier	6	M	Intact
Fox Terrier a pelo duro	6	F	Intact
Fox Terrier a pelo duro	5	M	Intact
Fox Terrier a pelo duro	4	F	Intact
Deutscher Jagd Terrier	2	M	Intact
West Highland White Terrier	4	M	Intact

West Highland White Terrier	2	F	Intact
Fox Terrier a pelo liscio	3	M	Intact
Fox Terrier a pelo duro	4	F	Intact
Parson Russell Terrier	5	M	Intact
Fox Terrier a pelo duro	1	M	Intact
Jack Russell Terrier	3	F	Intact
Jack Russell Terrier	11	F	Intact
Jack Russell Terrier	4	F	Intact
Jack Russell Terrier	8	F	Intact
Parson Russell Terrier	10	F	Intact
Jack Russell Terrier	9	F	Neutered
Jack Russell Terrier	2	M	Intact
West Highland White Terrier	4	M	Intact
West Highland White Terrier	13	F	Neutered
Scottish Terrier	7	F	Neutered
Scottish Terrier	6	F	Intact
Scottish Terrier	5	F	Intact
Scottish Terrier	2	F	Intact
Deutscher Jagd Terrier	4	M	Intact
Deutscher Jagd Terrier	2	F	Intact
Deutscher Jagd Terrier	4	F	Intact
Deutscher Jagd Terrier	8	M	Intact
Deutscher Jagd Terrier	13	F	Intact
Deutscher Jagd Terrier	4	M	Intact
Deutscher Jagd Terrier	3	F	Intact
Deutscher Jagd Terrier	5	M	Intact
Deutscher Jagd Terrier	10	M	Intact
Deutscher Jagd Terrier	12	F	Neutered
Deutscher Jagd Terrier	12	F	Neutered
Deutscher Jagd Terrier	1	M	Intact
Deutscher Jagd Terrier	4	F	Intact
Deutscher Jagd Terrier	3	F	Intact
Jack Russell Terrier	4	M	Intact
Jack Russell Terrier	6	F	Intact
Border Terrier	12	F	Neutered
Irish terrier	3	M	Intact
Terrier irlandese	4	M	Intact
Terrier Brasileiro	3	F	Intact
Jack Russell Terrier	1	F	Intact
English Toy Terrier Black and Tan	8	F	Intact
Jack Russell Terrier	17	F	Neutered
Parson Russell Terrier	7	M	Intact
Border Terrier	7	M	Intact
Jack Russell Terrier	2	F	Intact

West Highland White Terrier	1	M	Intact
Cairn Terrier	6	F	Intact
Jack Russell Terrier	3	M	Intact
Bedlington Terrier	4	F	Intact
Jack Russell Terrier	10	M	Intact
West Highland White Terrier	2	F	Intact
Jack Russell Terrier	11	F	Neutered
Boston terrier	1	M	Intact
Cairn Terrier	6	F	Intact
Cairn Terrier	4	F	Intact
Cairn Terrier	9	M	Intact
Cairn Terrier	13	M	Neutered
Cairn Terrier	11	F	Neutered
Cairn Terrier	8	F	Neutered
Fox Terrier a pelo duro	8	M	Intact
Fox Terrier a pelo duro	6	M	Intact
Fox Terrier a pelo duro	3	M	Intact
Fox Terrier a pelo duro	8	F	Neutered
Fox Terrier a pelo duro	5	F	Neutered
Irish soft coated wheaten terrier	6	F	Neutered
Irish Terrier	4	M	Intact
Irish terrier	6	F	Neutered
Jack Russell Terrier	2	F	Intact
Jack Russell Terrier	3	F	Intact
Jack Russell Terrier	7	F	Intact
Jack Russell Terrier	1	M	Intact
Jack Russell Terrier	7	M	Intact
Jack Russell Terrier	2	M	Intact
Jack Russell Terrier	2	M	Intact
Jack Russell Terrier	17	M	Intact
Jack Russell Terrier	9	F	Neutered
Jack Russell Terrier	12	F	Neutered
Jack Russell Terrier	12	F	Neutered
Jack Russell Terrier	1	F	Neutered
Jack Russell Terrier	15	F	Neutered
Jack Russell Terrier	13	F	Neutered
Jack Russell Terrier	5	M	Neutered
Jack Russell Terrier	7	M	Neutered
Jack Russell Terrier	4	M	Intact
Jack Russell Terrier	11	M	Intact
Jack Russell Terrier	7	F	Neutered
Jack Russell Terrier	12	M	Neutered
Jack Russell Terrier	10	M	Neutered
Parson Russell Terrier	5	F	Intact

Parson Russell Terrier	7	M	Intact
Parson Russell Terrier	7	F	Neutered
Parson Russell Terrier	14	F	Neutered
Parson Russell Terrier	9	F	Neutered
Scottish Terrier	9	F	Intact
Scottish Terrier	9	F	Intact
Scottish Terrier	5	M	Intact
Scottish Terrier	8	M	Intact
West Highland White Terrier	7	M	Intact
West Highland White Terrier	3	F	Intact
Jack Russell Terrier	7	M	Neutered
Jack Russell Terrier	7	M	Intact
Jack Russell Terrier	7	F	Intact
Parson Russell Terrier	5	F	Intact
Jack Russell Terrier	11	M	Intact
Jack Russell Terrier	13	F	Neutered
Jack Russell Terrier	4	F	Intact
Jack Russell Terrier	11	M	Intact
Jack Russell Terrier	11	M	Neutered
Scottish Terrier	1	F	Intact
Scottish Terrier	8	M	Intact
West Highland White Terrier	11	F	Intact
Parson Russell Terrier	10	F	Neutered
Jack Russell Terrier	10	M	Intact
West Highland White Terrier	11	M	Intact
Deutscher Jagd Terrier	6	F	Neutered
Deutscher Jagd Terrier	2	F	Intact
Deutscher Jagd Terrier	2	F	Intact
Deutscher Jagd Terrier	2	M	Intact
Deutscher Jagd Terrier	2	M	Intact
English Toy Terrier, Black and Tan	1	F	Intact
Fox Terrier a pelo duro	5	F	Intact
Irish Terrier	6	M	Intact
Irish Terrier	7	F	Neutered
Jack Russell Terrier	2	F	Intact
Jack Russell Terrier	14	F	Intact
Jack Russell Terrier	1	F	Intact
Jack Russell Terrier	9	M	Intact
Jack Russell Terrier	1	M	Intact
Jack Russell Terrier	8	F	Neutered
Jack Russell Terrier	7	F	Neutered
Jack Russell Terrier	3	F	Intact
Jack Russell Terrier	16	M	Intact
Jack Russell Terrier	10	F	Neutered

Jack Russell Terrier	8	F	Neutered
Jack Russell Terrier	10	F	Neutered
Parson Russell Terrier	4	M	Intact
Parson Russell Terrier	4	F	Neutered
Scottish Terrier	2	F	Intact
Scottish Terrier	2	M	Intact
Scottish Terrier	10	F	Neutered
Welsh Terrier	13	M	Intact
West Highland White Terrier	8	M	Intact
West Highland White Terrier	2	M	Intact
West Highland White Terrier	4	M	Intact
West Highland White Terrier	2	F	Intact
Jack Russell Terrier	1	F	Intact
Jack Russell Terrier	16	M	Intact
Deutscher Jagd Terrier	2	M	Intact
Deutscher Jagd Terrier	2	F	Intact
Deutscher Jagd Terrier	6	F	Neutered
Deutscher Jagd Terrier	2	F	Intact
Deutscher Jagd Terrier	2	M	Intact
Scottish Terrier	2	M	Intact
Jack Russell Terrier	12	M	Intact
Scottish Terrier	6	M	Intact

PARTICIPATION_IN_BREED_ACTIVITY	FREQUENCY	FREQUENCY_SESSIONS_MONTH	TRAINABILITY
Yes, breed-specific	Every two weeks	2.17	4.38
Yes, breed-specific	Occasionally / variable	0.55	4.13
Yes, breed-specific	Monthly	1.00	3.50
Yes, breed-specific	Monthly	1.00	3.50
Yes, breed-specific	Weekly	4.33	4.25
Yes, breed-specific	Weekly	4.33	3.63
Yes, breed-specific	Weekly	4.33	3.75
Yes, breed-specific	Occasionally / variable	0.55	3.75
Yes, breed-specific	Every two weeks	2.17	4.13
Yes, breed-specific	Weekly	4.33	3.63
Yes, breed-specific	Several times a week	7.40	3.13
Yes, breed-specific	Every two weeks	2.17	3.75
Yes, breed-specific	Weekly	4.33	4.13
Yes, breed-specific	Monthly	1.00	3.67
Yes, breed-specific	Monthly	1.00	3.38
Yes, breed-specific	Every two weeks	2.17	3.38
Yes, breed-specific	Monthly	1.00	4.13
Yes, breed-specific	Weekly	4.33	4.25
Yes, breed-specific	A few times a year	0.71	3.00
Yes, breed-specific	Monthly	1.00	3.13
Yes, breed-specific	Monthly	1.00	2.83
Yes, breed-specific	Monthly	1.00	3.75
Yes, breed-specific	Monthly	1.00	3.00
Yes, breed-specific	Monthly	1.00	3.38
Yes, breed-specific	Weekly	4.33	3.75
Yes, breed-specific	Every two weeks	2.17	3.50
Yes, breed-specific	Occasionally / variable	0.55	3.88
Yes, breed-specific	Every two weeks	2.17	3.43
Yes, breed-specific	A few times a year	0.13	3.25
Yes, breed-specific	Monthly	1.00	3.00
Yes, breed-specific	Monthly	1.00	4.00
Yes, breed-specific	Monthly	1.00	3.86
Yes, breed-specific	Monthly	1.00	4.50
Yes, breed-specific	A few times a year	0.17	2.71
Yes, breed-specific	Monthly	1.00	3.50
Yes, breed-specific	Occasionally / variable	0.55	3.13
Yes, breed-specific	Monthly	1.00	3.57
Yes, breed-specific	Occasionally / variable	0.55	3.71
Yes, breed-specific	Occasionally / variable	0.55	3.86
Yes, breed-specific	Several times a week	7.40	3.38
Yes, breed-specific	Several times a week	7.40	3.63

Yes, breed-specific	Occasionally / variable	0.55	3.00
Yes, breed-specific	Monthly	1.00	3.50
Yes, breed-specific	Weekly	4.33	3.13
Yes, breed-specific	Every two weeks	2.17	3.88
Yes, breed-specific	Monthly	1.00	3.57
Yes, breed-specific	Every two weeks	2.17	3.50
Yes, breed-specific	Monthly	1.00	3.50
Yes, breed-specific	Monthly	1.00	3.00
Yes, breed-specific	Monthly	1.00	3.88
Yes, breed-specific	Occasionally / variable	0.55	3.63
Yes, breed-specific	Occasionally / variable	0.55	3.00
Yes, breed-specific	Weekly	4.33	3.40
Yes, breed-specific	Weekly	4.33	4.00
Yes, breed-specific	Weekly	4.33	4.25
Yes, breed-specific	Every two weeks	2.17	3.63
Yes, breed-specific	Occasionally / variable	0.55	2.60
Yes, breed-specific	Occasionally / variable	0.55	3.57
Yes, breed-specific	Monthly	1.00	3.86
Yes, breed-specific	Weekly	4.33	4.00
Yes, breed-specific	Occasionally / variable	0.55	3.00
Yes, breed-specific	Every two weeks	2.17	3.88
Yes, breed-specific	Weekly	4.33	3.75
Yes, breed-specific	Weekly	4.33	3.50
Yes, breed-specific	Weekly	4.33	3.75
Yes, breed-specific	Monthly	1.00	3.17
Yes, breed-specific	Monthly	1.00	3.43
Yes, breed-specific	A few times a year	0.25	3.63
Yes, breed-specific	Monthly	1.00	2.71
Yes, breed-specific	Monthly	1.00	2.71
Yes, breed-specific	Weekly	4.33	3.50
Yes, breed-specific	Monthly	1.00	3.88
Yes, breed-specific	Monthly	1.00	3.29
Yes, breed-specific	Monthly	1.00	4.13
Yes, breed-specific	A few times a year	0.21	3.75
Yes, non-breed-specific	Daily		3.63
Yes, non-breed-specific	Several times a week		3.71
Yes, non-breed-specific	Monthly		3.88
Yes, non-breed-specific	Several times a week		3.88
Yes, non-breed-specific	Several times a week		3.25
Yes, non-breed-specific	Several times a week		4.00
Yes, non-breed-specific	Daily		3.88
Yes, non-breed-specific	A few times a year		3.75
Yes, non-breed-specific	Weekly		3.63
Yes, non-breed-specific	Several times a week		3.86

Yes, non-breed-specific	Weekly		3.63
Yes, non-breed-specific	Monthly		3.40
Yes, non-breed-specific	Several times a week		4.25
Yes, non-breed-specific	Several times a week		4.00
No			3.86
No			3.63
No			4.25
No			4.13
No			3.13
No			3.71
No			2.75
No			3.57
No			2.75
No			3.29
No			2.75
No			3.50
No			3.50
No			3.25
No			3.13
No			3.63
No			3.63
No			3.25
No			4.29
No			4.00
No			3.86
No			2.38
No			3.13
No			2.88
No			3.75
No			3.75
No			3.38
No			3.50
No			2.88
No			3.75
No			3.38
No			3.88
No			4.13
No			3.75
No			3.50
No			4.13
No			3.13
No			3.00
No			4.00
No			3.88

No			3.29
No			3.88
No			2.88
No			4.13
No			4.17
No			3.43
No			3.00
No			3.60
No			3.38
No			3.63
No			3.75
No			3.75
No			3.86
No			3.88
No			4.13
No			3.88
No			4.33
No			4.25
No			3.63
No			2.75
No			3.00
No			4.20
No			3.63
No			3.25
No			2.88
No			2.75
No			2.14
No			2.75
No			3.75
No			3.88
No			3.25
No			2.88
No			4.13
No			2.86
No			3.88
No			3.75
No			3.38
No			3.13
No			4.38
No			3.38
No			3.63
No			3.13
No			3.13
No			2.75

No			4.25
No			2.71
No			4.50
No			3.25
No			4.13
No			3.13
No			4.13
No			2.50
No			3.25
No			4.13
No			3.33
No			3.63
No			3.38
No			3.13
No			3.75
No			2.14
No			2.75
No			2.75
No			3.88
No			3.13
No			4.00
No			3.86

Aggression toward strangers	Aggression toward owner	Aggression toward dogs	Dog rivalry	Fear of strangers	Non-social fear	Dog directed fear
1.80	0.00	1.75	1.00	0.00	0.00	0.00
0.80	0.00	2.25	0.00	0.00	0.17	0.00
1.60	1.00	1.00	1.25	1.00	0.50	1.00
0.80	0.00	2.00	2.00	0.75	1.00	2.00
1.00	0.00	1.25	0.25	0.00	0.00	0.00
2.90	0.00	3.00	0.00	0.50	0.67	0.00
1.80	0.00	2.25	0.00	3.00	1.50	2.50
0.00	0.00	0.25	0.00	0.00	0.00	0.00
0.00	0.00	1.00	0.00	0.00	0.00	0.00
0.00	0.00	0.00	0.00	0.00	0.00	0.00
2.40	0.00	2.50	0.50	0.50	0.33	0.25
2.90	0.00	2.50	0.00	0.50	0.50	1.50
1.00	0.00	1.50	0.00	0.00	0.00	0.50
0.40	1.00	0.75	0.50	0.00	0.33	1.00
0.00	0.00	1.75	1.25	0.50	0.33	0.00
0.10	0.00	2.75	0.00	0.00	0.67	0.00
0.00	0.00	0.25	0.00	0.50	0.33	0.00
0.70	0.00	2.50	2.00	0.00	0.50	2.00
0.20	0.00	2.50	0.25	0.00	0.17	0.00
0.50	0.00	3.25	1.75	0.00	0.00	0.00
0.20	0.00	1.25	0.75	0.00	0.83	1.25
1.20	0.00	1.75	0.50	1.25	0.33	0.75
0.30	0.00	1.50	0.00	0.00	0.50	0.00
0.30	0.00	1.00	0.25	0.00	0.83	0.00
0.00	0.00	3.25	3.00	0.00	0.17	1.00
0.50	0.00	0.50	0.00	0.00	0.83	0.25
1.20	0.00	2.50	1.75	1.25	0.83	2.50
1.20	0.00	1.25	1.75	1.75	1.17	1.00
0.00	0.00	1.25	0.00	0.25	0.83	1.25
0.60	0.00	1.75	0.00	0.25	1.00	0.25
0.00	0.00	0.50	0.00	0.00	0.67	0.00
0.60	0.00	1.50	0.50	0.25	0.33	2.50
0.30	0.00	1.50	0.00	0.25	1.50	0.00
1.00	0.25	3.25	1.75	0.75	1.33	0.00
0.90	0.00	1.25	0.00	0.50	0.67	1.25
0.20	0.00	0.75	0.25	0.00	1.00	1.00
1.10	0.00	1.75	0.00	0.25	0.67	0.25
0.90	0.00	2.25	0.00	1.25	0.83	3.00
0.80	0.00	1.75	0.00	0.00	1.00	0.25
0.90	0.00	3.25	3.50	0.25	0.83	0.00
0.20	0.00	1.25	0.00	0.00	0.83	1.00

2.60	0.00	2.75	2.00	0.75	0.83	1.00
0.10	0.00	2.00	1.25	0.00	0.00	0.00
0.00	0.00	1.50	0.00	0.00	1.50	1.00
1.70	0.00	1.50	0.00	0.25	0.33	1.00
0.20	0.00	0.25	0.00	0.50	0.17	0.25
0.10	0.00	1.00	0.25	0.00	0.00	0.75
0.90	0.00	2.25	0.50	0.00	0.00	0.25
0.20	0.00	1.50	0.00	0.50	0.50	0.00
0.90	0.00	2.50	2.25	0.00	0.17	0.00
2.00	0.00	2.25	0.25	0.00	0.00	0.00
2.50	0.00	3.00	0.00	0.00	1.17	0.00
1.90	0.00	0.25	0.00	0.00	1.00	0.25
0.90	0.00	2.50	2.50	1.00	0.17	0.00
0.10	0.00	1.25	0.50	0.00	1.17	0.00
0.20	0.00	1.00	2.50	0.00	0.17	0.00
0.50	0.00	1.50	2.50	0.00	0.67	0.25
2.80	0.00	3.50	3.00	0.50	0.17	0.00
0.00	0.00	0.00	2.00	0.00	0.00	0.00
0.90	0.25	2.50	2.50	1.00	0.17	0.00
2.60	0.00	2.75	2.00	0.75	0.83	1.00
0.10	0.00	0.75	0.00	0.25	0.17	0.50
0.10	0.00	0.75	0.00	0.25	0.33	0.75
0.20	0.00	0.50	0.00	0.00	0.00	0.25
0.30	0.13	1.50	0.00	0.50	0.33	1.00
0.20	0.00	1.25	1.00	0.00	0.50	0.00
0.00	0.00	1.50	0.75	0.25	0.17	1.25
0.30	0.00	2.25	2.00	0.00	0.67	1.00
0.30	0.00	0.25	0.50	0.00	0.17	0.25
0.30	0.00	0.25	0.50	0.00	0.17	0.25
1.40	1.00	1.25	1.25	0.00	0.17	0.00
0.40	0.00	0.50	0.50	0.00	0.00	0.25
0.50	0.00	0.25	0.75	0.00	0.00	0.25
0.30	0.00	0.75	0.50	0.00	0.00	0.50
0.60	0.00	3.75	0.00	0.00	0.00	0.00
0.00	0.00	0.75	0.00	0.00	0.00	0.00
0.20	0.00	1.25	0.00	0.00	0.17	0.00
0.80	0.00	3.25	0.00	0.00	1.83	2.75
0.60	0.25	1.00	0.00	0.50	0.33	1.75
0.00	0.13	0.00	0.00	0.00	0.33	0.25
1.10	0.00	2.25	0.00	0.00	0.50	0.00
2.10	0.00	2.25	1.00	0.50	0.67	2.00
2.30	0.00	1.50	0.00	0.25	0.50	0.25
1.50	0.25	3.00	0.25	0.25	0.00	2.00
0.00	0.00	0.75	0.25	0.00	0.00	0.00

0.10	0.13	0.25	0.00	0.00	0.00	0.00
0.00	0.00	0.00	0.50	0.00	0.33	0.00
0.00	0.00	1.00	0.00	0.00	0.83	0.00
1.70	0.00	2.25	0.00	1.50	0.83	2.00
1.30	0.00	1.75	0.00	0.25	1.83	0.00
0.20	0.25	0.50	0.00	0.00	0.00	0.50
0.10	0.00	1.75	0.00	0.00	1.83	0.00
0.00	0.00	0.00	0.00	0.00	0.50	0.00
0.40	0.00	0.25	0.00	0.00	0.00	0.00
1.30	0.88	1.00	1.25	2.00	1.33	1.00
0.20	0.38	1.75	0.00	0.00	0.50	1.00
0.20	0.25	2.25	0.00	0.00	0.17	0.00
0.00	0.00	0.00	0.00	0.75	1.17	0.25
0.60	0.00	2.00	0.00	0.00	0.83	0.50
0.30	0.00	1.50	0.00	0.00	0.67	0.00
0.40	0.00	1.75	0.00	0.00	0.17	0.00
0.40	0.00	1.50	0.00	0.00	0.67	0.00
0.50	0.00	1.25	0.00	0.00	0.17	0.00
0.20	0.00	1.00	1.00	0.75	1.33	0.50
1.00	0.38	1.25	0.25	1.25	1.00	0.25
0.20	0.00	2.25	0.00	0.00	1.17	1.25
0.00	0.00	4.00	0.00	0.00	3.33	2.50
0.00	0.00	1.00	0.00	0.00	0.50	0.25
0.00	0.38	0.50	0.00	0.00	1.17	1.00
0.00	0.00	1.50	0.25	0.00	0.17	0.25
0.50	0.25	0.75	0.25	0.75	0.83	0.75
1.30	0.75	3.25	0.25	0.00	0.50	1.00
0.00	0.00	0.00	0.00	0.00	0.00	0.00
1.20	0.00	1.50	0.50	1.50	1.00	2.00
0.20	0.00	1.25	0.00	0.00	0.00	0.00
1.00	0.25	3.25	0.75	0.75	1.17	3.00
0.60	0.00	1.75	1.00	0.00	1.83	0.75
0.20	0.13	2.00	3.00	0.00	1.67	0.00
0.00	0.00	0.75	0.00	0.00	1.67	0.00
0.00	0.50	4.00	3.75	0.00	0.67	0.00
0.00	0.13	1.50	0.00	0.00	1.67	0.00
1.40	0.00	2.75	0.00	2.00	1.17	0.75
1.70	0.00	3.25	0.00	4.00	4.00	3.25
0.00	0.00	0.00	0.00	0.00	1.17	0.00
0.50	0.00	3.00	0.00	0.50	0.83	4.00
0.30	0.38	2.00	1.50	0.00	2.00	1.00
0.70	0.88	4.00	2.00	0.00	2.00	1.00
2.20	0.00	2.00	0.50	0.00	0.00	0.75
1.00	0.00	0.00	1.00	2.25	0.67	1.00

0.20	0.00	0.00	0.00	0.00	1.00	0.00
1.30	0.38	2.50	0.00	0.50	2.50	2.50
1.00	0.00	1.25	0.00	3.75	1.67	0.00
2.00	0.00	3.00	2.50	2.50	1.67	2.50
0.70	0.00	2.50	0.00	0.00	0.17	0.00
1.40	0.25	1.25	0.00	0.25	1.33	0.25
0.10	0.00	2.00	0.50	0.00	0.33	0.00
2.40	0.00	3.50	0.00	0.00	1.67	0.00
0.00	0.00	0.00	0.00	0.75	0.83	0.75
0.60	0.00	0.75	0.50	0.00	0.00	0.00
1.70	0.00	3.25	0.00	4.00	4.00	3.25
0.20	0.00	1.25	0.00	0.00	0.00	0.00
0.00	0.00	1.50	0.25	0.00	0.17	0.25
1.00	0.00	0.00	1.00	2.25	0.67	1.00
0.50	0.00	3.00	0.00	0.50	0.83	4.00
0.00	0.13	1.50	0.00	0.00	1.67	0.00
0.00	0.00	1.75	0.00	0.00	0.17	0.00
0.50	0.00	3.50	0.00	0.00	1.17	3.00
2.20	1.50	4.00	0.00	1.50	2.00	2.00
0.40	0.38	1.00	0.00	0.00	0.67	0.25
0.70	0.00	2.25	0.50	0.00	1.00	0.00
1.10	0.50	1.00	0.50	0.00	0.67	1.00
1.00	0.00	1.50	0.75	0.25	0.00	0.00
0.30	0.13	1.75	0.00	0.00	1.17	1.50
0.20	0.00	0.50	0.50	0.00	0.00	0.00
3.30	2.25	1.75	3.00	3.75	1.83	1.25
0.50	0.00	1.00	1.00	0.75	0.83	1.25
0.40	0.13	1.50	0.75	0.00	0.83	1.50
0.00	0.00	0.25	0.00	0.75	1.50	3.00
0.90	0.00	1.00	0.00	1.50	1.00	2.50
0.10	0.00	0.25	0.00	0.75	0.33	0.50
1.80	1.25	3.75	4.00	0.00	0.00	0.00
1.20	0.63	2.75	0.00	0.25	1.00	0.50
0.10	0.00	1.50	0.00	0.00	0.00	0.75
0.00	0.00	0.00	0.00	0.00	1.00	0.50
0.10	0.00	1.75	1.25	0.00	0.00	1.75
0.30	0.13	0.75	0.50	0.00	0.83	1.00
0.60	0.25	1.75	0.75	0.00	0.33	1.25
0.00	0.00	0.00	0.00	0.00	0.17	0.00
0.00	0.00	0.50	1.50	2.00	3.33	2.75
0.20	0.00	2.50	0.00	0.00	1.00	0.00
0.20	0.00	0.75	3.00	1.50	0.67	2.00
0.80	0.13	1.25	0.00	0.00	1.00	1.00
1.80	2.13	1.75	2.50	0.00	0.83	2.25

0.40	0.00	1.75	0.00	0.25	0.67	0.50
1.10	0.75	2.25	1.25	0.50	1.67	1.00
0.70	0.00	1.50	0.00	2.00	2.67	1.25
0.70	0.00	0.75	0.00	0.25	1.00	0.00
1.00	0.63	1.00	0.00	0.00	1.00	0.50
1.30	0.13	2.00	0.75	0.25	1.17	0.75
0.50	0.50	2.50	2.50	1.00	3.17	1.00
0.30	0.50	3.00	3.00	0.00	0.50	0.00
0.00	0.13	1.00	2.00	0.50	0.00	0.00
0.40	0.00	1.50	0.00	0.25	1.50	1.50
0.20	0.00	2.00	1.50	0.00	0.50	0.00
1.80	2.63	0.25	0.00	1.00	1.00	0.50
0.30	0.13	0.75	0.50	0.00	0.83	1.00
0.80	0.13	1.25	0.00	0.00	1.00	1.00
0.00	0.00	0.25	0.00	0.75	1.50	3.00
0.50	0.00	1.00	1.00	0.75	0.83	1.25
3.30	2.25	1.75	3.00	3.75	1.83	1.25
0.40	0.13	1.50	0.75	0.00	0.83	1.50
0.90	0.00	1.00	0.00	1.50	1.00	2.50
1.30	0.13	2.00	0.75	0.25	1.17	0.75
0.90	0.00	1.25	0.50	0.50	0.67	1.00
1.20	0.25	2.50	0.00	0.00	1.33	0.00

Separation-related behaviors	Excitability	Attachment/ attention-seeking	Touch sensitivity	Energy level	Chasing behavior	Miscellaneous behaviors
1.75	2.8	4.33	0.25	4.00	4.75	1.82
1.00	1.5	2.67	0.00	1.00	4.50	1.23
2.43	1.8	4.00	0.00	4.00	3.50	2.25
1.13	2.5	3.17	0.50	1.50	4.50	2.27
1.25	1.8	3.50	0.00	3.50	4.75	1.55
3.88	3.3	4.80	1.00	4.00	4.75	2.64
1.63	3.7	4.00	1.25	2.50	3.75	1.64
1.63	1.0	3.00	1.25	2.50	4.50	1.45
1.13	1.0	2.83	0.75	2.00	3.50	1.05
1.50	3.4	4.00	0.00	4.00	2.25	1.27
1.38	3.3	3.83	0.50	4.50	4.50	1.73
1.13	2.2	4.83	1.00	3.00	4.00	1.95
1.13	1.0	3.17	0.00	2.50	4.50	1.41
1.13	1.0	3.33	0.00	5.00	3.00	1.71
1.00	2.0	2.33	0.25	3.00	4.25	1.32
1.25	3.4	4.50	0.00	4.50	4.67	2.18
1.38	3.3	2.83	0.00	4.00	5.00	1.41
1.75	1.7	3.00	0.00	3.50	4.25	1.77
1.38	2.7	2.33	0.00	1.00	4.00	1.64
1.00	1.3	2.17	0.00	4.50	4.50	1.36
1.57	1.4	1.60	0.75	2.00	3.50	2.00
1.38	1.8	2.67	0.75	3.50	3.00	1.36
1.00	1.3	2.83	0.00	4.00	4.50	1.27
1.00	1.7	2.67	0.00	4.00	4.50	1.27
1.25	3.2	2.50	0.25	3.00	4.75	1.36
1.50	1.5	3.83	0.25	3.00	4.75	2.27
2.00	1.8	2.67	0.00	2.00	4.50	1.38
1.25	1.8	2.33	0.25	2.50	4.50	1.41
1.00	1.0	3.00	0.75	3.00	4.25	1.36
1.00	2.5	3.67	0.00	4.00	3.75	1.50
1.13	3.3	1.67	0.00	3.50	5.00	1.36
1.50	1.8	3.50	1.75	3.00	4.75	1.41
2.13	2.8	3.80	1.50	3.50	4.75	1.95
1.25	1.4	2.67	1.25	1.00	4.75	1.41
1.13	2.0	3.83	0.75	3.50	2.00	1.41
1.75	2.5	2.67	0.25	3.50	4.75	2.33
1.83	3.0	3.17	1.00	1.00	3.50	1.36
1.25	3.8	4.17	0.75	4.50	4.00	1.52
1.00	2.2	2.17	0.00	2.00	3.75	1.64
1.38	2.4	2.83	0.75	3.50	3.33	1.67
1.00	2.0	1.67	0.00	3.00	4.50	1.50

1.88	3.2	4.00	1.25	4.00	4.75	1.64
1.25	2.7	4.00	0.50	5.00	4.50	1.90
1.00	1.8	2.50	1.75	2.50	4.75	1.68
1.25	1.3	3.50	0.50	1.00	4.75	1.55
1.25	1.0	2.67	0.25	2.00	3.25	1.36
1.38	3.7	3.20	0.00	4.00	4.75	1.18
1.00	1.8	2.67	0.50	3.00	2.75	1.41
1.88	2.4	4.17	0.25	3.00	4.75	2.27
1.71	1.0	3.33	0.75	2.50	4.75	1.45
1.75	3.0	2.50	0.00	4.00	4.75	1.68
1.50	3.0	2.33	0.00	2.00	4.50	1.23
1.75	3.6	4.00	0.50	3.00	4.75	1.71
1.13	1.2	2.67	0.00	4.50	4.75	1.50
1.00	1.2	3.00	0.75	2.50	4.75	1.36
1.13	2.0	1.33	2.00	1.00	4.00	1.50
1.63	2.7	3.33	2.50	2.50	4.75	1.82
1.38	2.8	3.33	2.25	3.00	4.75	1.45
2.00	2.4	2.17	0.00	3.00	4.50	1.32
1.13	1.2	2.67	0.00	4.50	4.75	1.50
1.88	3.2	4.00	1.25	4.00	4.75	1.64
1.75	3.0	4.00	0.50	4.00	2.25	0.86
1.00	2.8	2.33	0.50	3.50	1.75	1.00
1.00	2.5	2.60	0.50	4.00	1.67	0.91
1.25	3.0	3.00	0.50	3.00	1.75	1.00
1.88	2.0	3.00	1.00	3.00	4.33	1.65
1.38	1.8	2.33	0.25	2.50	4.50	1.38
1.50	3.0	4.33	2.00	4.00	4.25	2.05
1.00	1.0	1.50	0.50	1.00	2.50	1.45
1.00	1.0	1.50	0.50	1.00	2.50	1.45
1.63	2.2	3.17	0.25	4.00	4.25	1.82
1.13	1.5	3.00	0.00	5.00	4.25	1.32
1.25	1.2	3.00	0.00	3.50	4.00	1.23
1.25	1.2	2.17	0.00	5.00	3.75	1.50
1.13	1.5	2.67	0.00	3.50	4.75	1.36
2.50	2.5	2.33	0.25	2.00	1.33	1.50
1.00	1.3	3.67	0.00	3.00	4.25	1.91
1.50	4.0	4.50	1.50	3.50	4.00	1.82
1.50	2.3	2.83	0.00	5.00	3.50	1.68
1.38	1.0	2.80	0.00	4.50	3.25	1.36
1.75	4.0	3.83	0.50	3.00	1.67	1.68
1.88	2.7	3.67	1.00	5.00	1.00	1.55
2.13	4.0	4.50	0.00	4.50	4.75	2.32
1.50	2.0	3.50	0.25	2.50	3.50	1.32
1.00	2.4	3.50	0.00	5.00	4.75	1.41

1.25	2.2	3.50	0.00	4.00	2.67	1.59
1.25	4.0	2.17	0.75	3.50	4.33	1.14
1.13	2.8	3.40	0.25	4.00	4.50	1.57
1.13	2.8	2.17	0.50	3.00	4.75	2.10
1.86	3.0	4.33	0.50	4.00	3.75	2.09
1.00	2.8	4.50	0.75	2.50	4.00	1.36
1.00	2.3	3.67	0.00	2.00	1.75	1.09
1.00	2.8	2.00	0.00	3.00	4.00	1.18
1.00	1.0	2.33	0.00	2.00	4.50	1.55
2.13	1.0	3.50	1.25	4.00	2.00	2.05
1.63	1.8	2.33	1.00	3.00	4.00	2.18
1.00	1.0	2.50	0.50	1.50	1.25	1.05
1.75	2.0	3.33	0.00	1.00	4.00	1.82
1.00	2.0	3.33	1.75	2.00	4.75	1.86
1.00	1.4	2.67	0.25	4.50	3.75	1.32
1.00	1.0	2.83	0.25	4.50	4.50	1.32
1.13	1.2	3.33	0.50	4.50	4.25	1.32
1.00	1.3	2.50	0.25	3.00	4.50	1.36
1.25	1.5	4.00	1.25	4.50	3.50	1.41
3.00	3.2	4.17	1.00	1.50	1.67	1.95
1.50	2.3	2.50	0.25	4.00	2.50	1.65
1.50	2.5	3.67	2.50	3.00	4.50	1.91
2.00	2.4	3.00	0.25	5.00	4.25	2.27
1.13	3.6	3.00	1.50	4.00	3.50	2.09
1.38	1.2	3.00	0.25	1.00	4.50	1.23
2.57	2.3	4.17	0.50	4.00	4.50	1.57
1.88	2.8	4.67	1.25	3.50	4.50	2.36
1.25	2.3	2.00	0.00	3.00	3.25	1.73
1.63	3.7	4.00	0.00	5.00	2.00	2.09
1.25	1.5	1.33	0.00	3.00	4.50	1.52
1.00	2.3	3.33	1.00	4.00	4.00	1.95
1.88	3.2	4.33	0.75	2.50	4.75	2.59
1.00	2.0	4.17	1.00	3.00	4.50	1.82
1.00	2.6	2.33	0.50	2.00	3.75	1.64
1.00	3.5	2.83	0.75	5.00	3.75	2.14
1.20	2.5	3.67	1.25	3.50	2.00	1.95
1.38	2.8	5.00	0.75	3.50	4.75	2.27
1.00	2.6	2.83	1.50	1.50	1.75	1.77
1.25	2.8	4.20	1.50	5.00	1.75	1.18
1.88	2.2	5.00	0.00	4.00	2.50	1.23
1.25	2.3	5.00	2.75	4.00	4.75	2.55
2.75	3.5	2.83	0.50	5.00	4.75	3.00
1.00	3.2	3.33	0.50	3.50	4.75	1.74
1.38	2.3	4.83	0.50	2.00	4.50	1.91

1.13	2.7	3.00	0.00	4.00	4.25	1.55
1.63	2.0	2.67	0.75	4.50	4.00	1.82
1.75	2.4	4.20	1.25	2.00	4.75	2.14
1.63	4.0	3.00	0.00	2.00	4.75	1.55
1.13	1.0	2.83	0.00	2.50	2.75	1.57
1.50	1.5	2.83	1.75	1.50	4.00	1.64
1.38	4.0	4.67	0.25	1.00	4.67	1.45
1.00	2.0	3.00	0.75	1.50	4.00	2.00
1.00	1.7	3.83	1.00	1.50	3.00	1.73
1.38	1.0	3.50	0.25	4.00	3.00	1.50
1.00	2.6	2.83	1.50	1.50	1.75	1.77
1.25	1.5	1.33	0.00	3.00	4.50	1.52
1.38	1.2	3.00	0.25	1.00	4.50	1.23
1.38	2.3	4.83	0.50	2.00	4.50	1.91
1.88	2.2	5.00	0.00	4.00	2.50	1.23
1.20	2.5	3.67	1.25	3.50	2.00	1.95
1.00	3.0	2.60	0.00	2.00	4.00	1.50
1.88	4.0	3.50	0.00	3.50	4.00	1.64
1.38	3.0	3.67	0.50	3.50	4.75	1.50
1.75	2.3	3.50	1.25	2.00	4.75	1.86
1.63	2.0	2.67	0.75	2.50	3.00	1.82
1.25	3.5	4.67	0.75	4.00	1.00	1.77
1.13	1.5	3.50	0.25	2.00	3.00	1.32
1.00	2.3	3.00	1.25	3.00	1.67	1.64
1.00	2.7	3.83	0.00	1.00	4.75	1.64
1.75	2.0	3.50	2.00	2.50	4.00	2.05
2.13	2.8	1.50	1.00	3.50	3.00	1.36
1.75	1.8	2.50	0.75	2.00	2.25	1.27
3.00	3.0	3.80	1.00	5.00	2.00	1.33
1.83	3.2	4.00	0.50	5.00	3.50	1.50
1.00	1.5	3.00	1.00	3.00	3.67	1.23
3.13	3.8	1.00	0.00	4.00	4.50	1.82
2.75	2.8	4.00	1.50	4.00	4.75	1.68
1.13	1.0	2.00	0.00	3.00	3.00	1.91
1.63	3.2	2.33	0.00	4.50	4.67	1.95
2.75	2.4	3.33	0.00	1.00	2.75	1.55
1.25	3.8	5.00	0.25	4.50	4.50	2.14
1.38	2.5	3.50	1.00	3.00	3.75	1.86
1.00	2.0	3.50	0.00	5.00	2.00	1.59
2.25	2.2	3.33	0.50	2.00	3.00	2.23
1.00	3.2	4.20	0.00	4.00	4.67	1.52
2.25	2.5	5.00	0.25	3.00	3.00	2.00
1.25	2.5	4.83	2.00	3.50	4.50	2.00
3.13	3.5	3.67	3.25	4.00	4.25	1.73

2.63	2.8	4.17	0.75	5.00	3.25	1.82
2.00	3.2	2.83	0.00	1.50	3.75	1.52
1.25	3.5	4.33	1.00	5.00	5.00	1.91
1.50	1.5	3.33	2.25	4.00	4.00	2.00
2.00	2.4	3.83	0.25	4.50	3.00	1.45
1.40	2.3	2.83	1.00	4.00	2.75	1.38
1.25	3.5	2.67	1.25	2.50	3.75	2.23
1.63	1.3	1.50	0.25	3.00	4.75	1.73
1.13	1.5	2.83	0.50	1.00	3.00	1.45
2.63	3.3	3.83	2.00	4.00	5.00	2.57
1.14	2.3	4.33	0.75	4.00	4.50	1.58
2.00	3.5	2.83	2.75	4.00	5.00	2.27
1.25	3.8	5.00	0.25	4.50	4.50	2.14
1.25	2.5	4.83	2.00	3.50	4.50	2.00
3.00	3.0	3.80	1.00	5.00	2.00	1.33
2.13	2.8	1.50	1.00	3.50	3.00	1.36
1.75	2.0	3.50	2.00	2.50	4.00	2.05
1.75	1.8	2.50	0.75	2.00	2.25	1.27
1.83	3.2	4.00	0.50	5.00	3.50	1.50
1.40	2.3	2.83	1.00	4.00	2.75	1.38
1.00	1.8	4.00	0.00	1.00	2.33	1.36
1.00	2.2	1.67	0.75	2.00	3.75	1.09
